# Modification and validation of the COVID-19 stigma instrument in nurses: A cross-sectional survey

**DOI:** 10.3389/fpsyg.2023.1084152

**Published:** 2023-08-18

**Authors:** Feifei Huang, Wenxiu Sun, Yonglin Li, Lin Zhang, Wei-Ti Chen

**Affiliations:** ^1^School of Nursing, Fujian Medical University, Fuzhou, China; ^2^Shanghai Public Health Clinical Center, Fudan University, Shanghai, China; ^3^School of Nursing, University of California, Los Angeles, Los Angeles, CA, United States

**Keywords:** COVID-19, stigma, nurses, psychometrics, survey

## Abstract

**Background:**

Nurses taking care of patients with infectious diseases have suffered from noticeable societal stigma, however currently, there is no validated scale to measure such stigma. This study aimed to revise and validate the COVID-19 Stigma Instrument-Nurse-Version 3 (CSI-N-3) by using item response theory (IRT) as well as classical test theory analysis.

**Methods:**

In phase I, the Chinese CSI-N-3 was modified from the English version of HIV/AIDS Stigma Instrument-Nurse based on standard cross-cultural procedures, including modifications, translation/back translations, pilot testing, and psychometric testing with classical test theory and Rasch analysis. In phase II, a cross-sectional study using cluster sampling was conducted among 249 eligible nurses who worked in a COVID-19-designed hospital in Shanghai, China. The influencing factors of COVID-19-associated stigma were analyzed through regression analysis.

**Results:**

In phase I, the two-factor structure was verified by confirmatory factor analysis, which indicated a good model fit. The 15-item CSI-N-3 achieved Cronbach’s *α* of 0.71–0.84, and composite reliability of 0.83–0.91. The concurrent validity was established by significant association with self-reported physical, psychological, and social support levels (*r* = −0.18, −0.20, and −0.21, *p* < 0.01). In IRT analysis, the CSI-N-3 has ordered response thresholds, with the Item Reliability and Separation Index of 0.95 and 4.15, respectively, and the Person Reliability and Separation Index of 0.20 and 0.50, respectively. The infit and outfit mean squares for each item ranged from 0.39 to 1.57. In phase II, the mean score for the CSI-N-3 in Chinese nurses was 2.80 ± 3.73. Regression analysis showed that social support was the only factor affecting nurses’ COVID-19-associated stigma (standardized coefficients *β* = −0.21, 95% confidence interval: −0.73 ~ −0.19).

**Conclusion:**

The instrument CSI-N-3 is equipped with rigorous psychometric properties that can be used to measure COVID-19-associated stigma during and after the COVID-19 pandemic among nurses. The use of this instrument may facilitate the evaluation of tailored stigma-reduction interventions.

## Introduction

The coronavirus disease 2019 (COVID-19) global pandemic (World Health Organization, 2020) has placed frontline healthcare workers (HCWs) under extraordinary stress related to the high risk of infection, and resultant understaffing, uncertainty, and psychological distress (e.g., anxiety, depression, or insomnia) related to the illness (Cai et al., 2020; Liu et al., 2020). This is especially true for nurses, who make up the largest group of HCWs and who spend long periods of time providing care and monitoring COVID-19 patients (Chen et al., 2019). Nurses are often directly exposed to the virus and therefore are at high risk of developing the disease (Chen et al., 2019; Fernandez et al., 2020; Mo et al., 2020).

Compared with HIV, hepatitis, influenza A, H1N1, severe acute respiratory syndrome (SARS), and Middle East respiratory syndrome (MERS; Park et al., 2018; Nyblade et al., 2019; Chersich et al., 2020), COVID-19 is highly communicable and has higher mortality rates, with stigma figuring prominently among nurses working with COVID-19 patients. Studies estimate approximately 20–49% of nurses in Taiwan and Singapore experienced social stigmatization during the SARS outbreak (Bai et al., 2004; Koh et al., 2005), with one such example being a nurse who was scolded by fellow passengers for making trains “dirty” (Chersich et al., 2020). During 2015 MERS pandemic, while caring for MERS patients, Korean nurses were discriminated against by family members, friends, and neighbors as well as by community members in the schools that their children attended (Jung and Cho, 2015).

During the recent COVID-19 pandemic, several studies have reported that stigma has been experienced by HCWs. In one study by Simeone et al., Italian nurses experienced “stigma in the working environment” and “stigma in everyday life” (Simeone et al., 2021). Echoed in another study, Egyptian physicians experienced stigma while taking care of COVID-19 confirmed cases (Mostafa et al., 2020). Similarly, healthcare providers in Iran have also been impacted by COVID-related stigma (Kalateh Sadati et al., 2021). One study on perceptions of HCWs during the COVID-19 pandemic, conducted with non-healthcare worker adults, showed that study participants feared and avoided interactions with healthcare workers. This is a wide-spread and under-recognized issue during the COVID-19 pandemic (Taylor et al., 2020). These reports provide evidence that during the COVID-19 outbreaks, nurses have suffered from COVID-19-associated stigma due to the contagious nature and serious and potentially deadly outcomes of the disease (Bruns et al., 2020). COVID-19 related stigmatization among HCWs has been reported globally. For example, 17.3–91.0% of HCWs in Egypt experienced COVID-19-related stigma (Brooks et al., 2020). In addition, HCWs complained about their personal experiences with discrimination and later, burned out from caring for COVID-positive patients (Shiu et al., 2022). However, there is a dearth of empirical data on the measurement of COVID-related stigma experienced specifically by nurses.

The lack of research regarding COVID-19-associated stigma is due to the unavailability of validated measures of such stigma. Most measures used to explore the stigma of HCW during SARS (Ho et al., 2005), influenza A, and H1N1 (Kisely et al., 2020) were informal assessments that were not evaluated for reliability and validity. Several existing instruments are currently being used to measure the HIV/AIDS-related stigma of people living with HIV, the general population, and HCWs’ perceived stigma while taking care of HIV-infected individuals (Holzemer et al., 2009; Uys et al., 2009).

For this study, we adopted the HIV Dynamic Model of Holzemer et al. (2007) as the theatrical framework to guide the development of the stigma process and adapted the COVID-19 Stigma Instrument-Nurse (CSI-N) as a tool to measure levels of perceived stigma in nurses. The model is equipped three components, including the healthcare system, the environment (culture, economics, politics, law, and policies) and the agents (person, family, workplace, and community). The stigma process includes stigma triggers (testing, diagnosis, disease, disclosure, and suspicion), stigmatizing behaviors (blame, insult, avoidance, and accusation), types of stigmas (received, internal and associated), and stigma outcomes (poorer health, decreased quality of life, denied access to care, violence, and poorer quality of work life; Holzemer et al., 2007).

The 19-item HIV/AIDS stigma instrument-nurse (HASI-N) scale was the first reliable and valid scale used to measure HIV/AIDS-related stigma that is perpetrated and experienced by nurses; it includes two domains—nurses stigmatizing patients and nurses being stigmatized (Brooks et al., 2020). As the authors noted, the HASI-N scale could be modified to address infectious diseases other than HIV/AIDS, and considering similar stigma conditions may be experienced by HCWs who provide care to individuals with COVID-19, these authors felt it appropriate to modify the HASI-N for use in COVID-19. The COVID-19 Stigma Instrument-Nurse (CSI-N) scale was designed to measure COVID-19–related stigma among nurses.

High perceived stigma is directly associated with worse mental health among HCWs caring for HIV patients in Africa (Uys et al., 2009), MERS patients in Korea (Park et al., 2018), and SARS patients in Singapore (Verma et al., 2004), but findings regarding the linkage of stigma to HCWs’ physical health outcomes are mixed (Uys et al., 2009; Park et al., 2018; Logie and Turan, 2020). Perceived stigma may impair nurses’ job satisfaction and decrease their ability to provide effective care and therefore undermine the quality of care they provide (Chang and Cataldo, 2014; Nyblade et al., 2019). However, the stigma experienced by nurses during the COVID-19 pandemic and its influencing factors is still unknown. Limited studies have shown that COVID-19-infected individuals presenting with anxiety, higher levels of education, perceived risks, and familiarity with quarantine policy have a high likelihood of perceived stigma (Duan et al., 2020). Thus, in how to measure stigma associated with COVID-19 and its effects, a validated, trustworthy, and effective method was required to assess both the levels of stigma experienced by nurses during the pandemic as well as its influencing factors. In this paper, we present how we (1) modified the HASI-N into the CSI-N, (2) validated the CSI-N with both classical test theory (CTT) and item response theory (IRT), and (3) report on the COVID-19-associated stigma experienced by frontline nurses and its influencing factors.

## Methods

### Participants and setting

A convenience sampling method was used in Shanghai, China to recruit 400 Chinese registered nurses working in a COVID-19-designated facility. Two hundred and forty-nine eligible Chinese registered nurses participated. Nurses were eligible to participate if they rotated through COVID wards, understood the purpose of the survey, and were willing to complete the survey. The ratio of subjects to variables determined the sample size of 5–10 to 1, (Streiner and Norman, 2003) and yielded a total of 11 variables in the study survey. All participants were reimbursed after completing the survey.

### Design and procedure

After the approval of the study by the relevant institutional ethical review boards, our study took a two-stage approach that included a Stage I instrument modification and validation, and a Stage II cross-sectional survey.

#### Stage I: Instrument modification and validation

In this stage, we adhered to the COnsensus-based Standards for the selection of health status Measurement INstruments (COSMIN) checklist (Mokkink et al., 2010a,b). The HASI-N (Uys et al., 2009) comprises 19 questions/items and two factors: nurses stigmatizing patients (e.g., “*A nurse provided poorer quality care to an HIV/AIDS patient than to other patients*”) and nurses being stigmatized (e.g., “*People said nurses who provide HIV/AIDS care are HIV-positive*”). A four-point Likert scale ranging from 0 = “never” to 3 = “most of the time” was used to measure these questions/items. A Cronbach’s alpha reliability of 0.90 was calculated in this HASI-N scale showing good reliability. A negative association between job satisfaction and stigma significantly reinforced the HASI-N construct validity (Uys et al., 2009).

**Figure 1:**
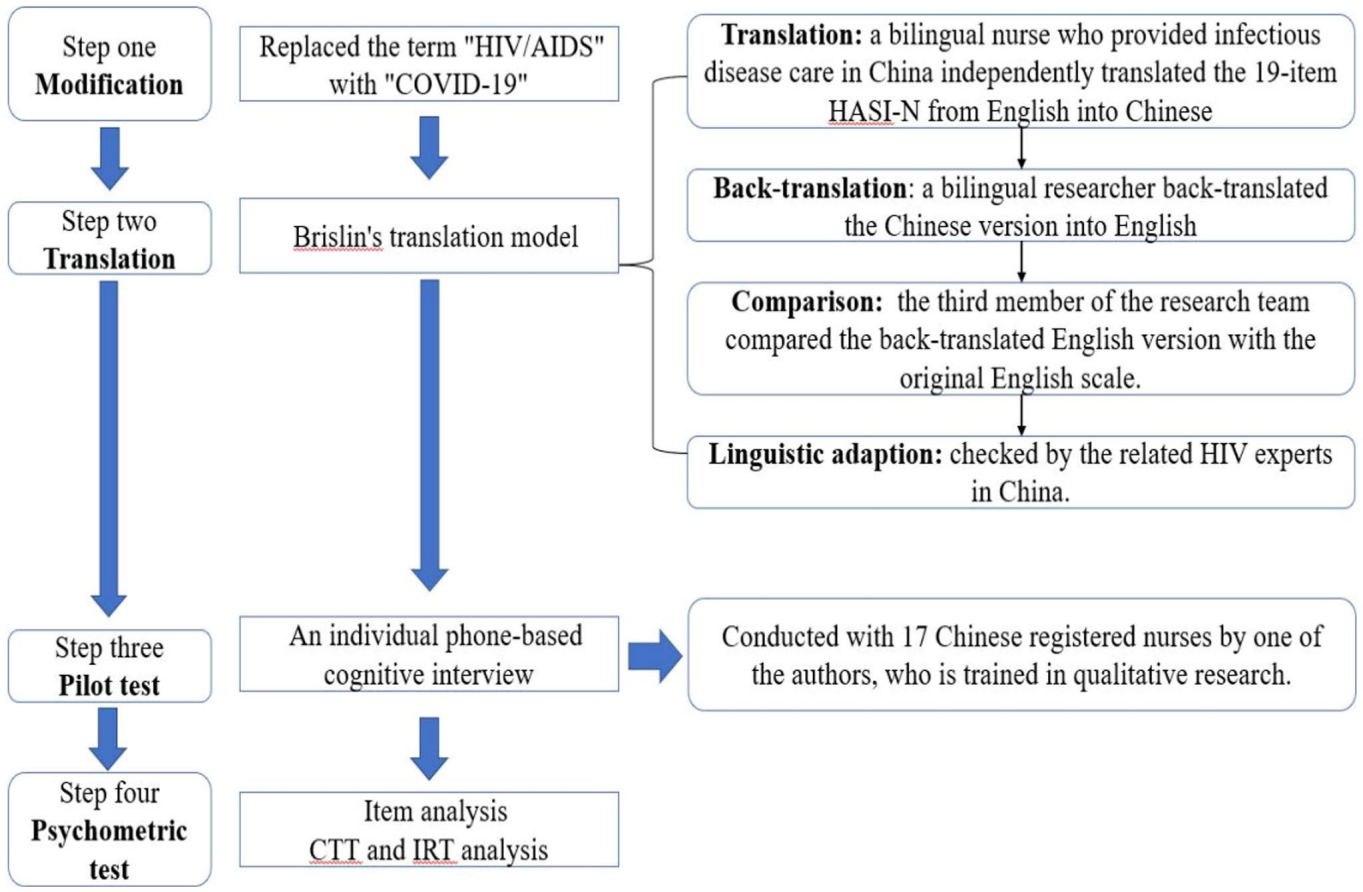
The cross-cultural adaption processes the HIV/AIDS stigma instrument-nurse (HASI-N) to COVID-19 stigma instrument-nurse (CSI-N). CTT, classical test theory; IRT, item response theory.

After obtaining permission from the original author of HASI-N, we revised the HASI-N into the CSI-N using four steps (see Figure 1).

##### Step 1: Modification

We use “COVID-19” instead of “HIV/AIDS” in the CSI-N.

##### Step 2: Translation

The translation model was followed during the trans-cultural interpretation of the HASI-N with a sequence of (1) translation, (2) back-translation, (3) comparison, and (4) linguistic adaption (Brislin, 1970; Jones et al., 2001). First, the 19-item HASI-N was translated by a bilingual nursing researcher (English to Chinese). Also, the back-translation of the Chinese into English version was done by another bilingual researcher, followed by a third member who compared the back-translated English version with the first version of the English instrument. One question was revised to confirm the two versions (translation and back-translated) close to the first (original)-version. Specifically, the Chinese sentence “*直呼护士的名字*” (“Someone called a nurse names”) in item 15 was replaced with “*耻笑护士*” (“scorn nurse”). The process resulted in the first version (version 1) of the Chinese Stigma Instrument-Nurse–Nurse (CCSI-N) to (CCSI-N-1) for pilot testing.

##### Step 3: Pilot test

To ensure fluency, readability, as well as comprehensibility of the new scale, one-on-one interviews were conducted with 17 nurses by phone and used a structured interview guide to understand how nurses translated items of the CCSI-N-1. Probes included: “Tell me what is this question asking?”; “What answer would you give to this question?”; and “What does the [survey concept] mean to you?” All interviews were digitally recorded and transcribed verbatim for later analysis. The nurses indicated that the description of item 7 (*A nurse made a COVID-19 patient wait until the last for care*) was not suitable considering the centralized treatment and care for COVID-19 patients; therefore, we deleted that item. The 18-item Chinese version 2 of CSI-N (CCSI-N-2) was ready for the validation steps.

##### Step 4: Psychometric test

The CTT and IRT evaluate the psychometric properties of the scale. After completing the item analysis, three items/questions (I5, I12, and I14) were discarded, therefore, the final 15 items/questions of CCSI-N-3 were generated (see Supplementary material A).

#### Stage II: Cross-sectional survey

In the cross-sectional survey, we adhered to the Strengthening the Reporting of Observational Studies in Epidemiology statement (von Elm et al., 2014). From April 18 to May 23, 2020, data were collected using the Questionnaire Star (QS or Wenjuanxing), an online survey program in China similar to the US-based Survey Monkey. We posted study recruitment information during the monthly nurse meetings at the COVID-19 facility. If eligible nurses were interested in participating and able to provide informed consent for the study online, the QR code or URL for the CCSI-N-3 was shared *via* the online messaging/calling system We Chat.^1^ Eligible nurses self-administered the 15-min online survey, including standardized measures to collect demographic data, self-reported health status, and social supports, as well as the CCSI-N-3. The sociodemographic variables included participants’ age, gender, marital status, ethnicity, highest educational level, professional title, and years as a nurse. The self-reported physical health, psychological health, and social support levels were measured by three questions; each of these factors was rated on a 10-point Likert scale from 1 = “very bad” to 10 = “very well.” In this study, the social support construct assessed nurses’ support from family, colleagues, or the hospital they worked for.

### Data analysis

SPSS 23.0 (IBM, Chicago, IL, United States) and Mplus 6.1 (Muthén & Muthén, Los Angeles, CA, United States) were used for data analyses. In addition, IRT analysis used WINSTEPS 3.75.0 (Chicago, IL, United States) for the final report; *p* < 0.05 was considered significant.

#### Stage I: Statistical analysis

##### Item analysis

The item was deleted when it met the following criteria of CTT and IRT analysis: (1) factor loading or cross-loading <0.4; (2) infit and outfit mean squares over the range of 0.6–1.4; and (3) after items were deleted, the alpha coefficient for the overall scale was increased (Nguyen et al., 2014; Huang et al., 2017).

##### Structural validity

The structural validity of the scale was assessed by CTT and IRT analyses combined with the confirmatory factor analysis (CFA). In the CFA, the best-fitting model of the scale was examined by using the method of maximum likelihood. The model’s goodness of fit was evaluated with normed *χ*^2^ (*χ*^2^/df) between 1.0 and 3.0, Root Mean Square Error of Approximation (RMSEA), Comparative Fit Index (CFI), Tucker-Lewis Index (TLI), and Normed Fit Index (NFI; Johnson et al., 2011; Huang et al., 2017).

In the IRT analysis, we examined the unidimensionality assumptions of the IRT analysis by a principal component analysis (PCA). Assuming nurses may interpret scales differently in terms of the items, we used the partial credit model to assess the item and person separation reliability, item and person separation index, category probability curves, infit and outfit mean squares, test information function (TIF; Baker, 2001), and differential item functioning (DIF; Campbell and Fiske, 1959; Wolfe and Smith, 2007).

##### Convergent validity

*Convergent validity* of the CSI-N-3 was estimated by computing Pearson’s correlations between the CSI-N-3 and self-reported physical health, psychological health, and social support levels.

##### Internal consistency reliability

*Internal consistency reliability* was estimated by Cronbach’s *α* coefficient (Ahorsu et al., 2020).

#### Stage II: Statistical analysis

The one-sample Kolmogorov–Smirnov tests were not statistically significant even though the data fitted the assumptions of normality. Continuous variables were presented as standard deviations (SDs) and mean. Categorical variables were presented as percentages and means or proportions and SDs. Then, this followed up with one-way ANOVA and independent *t*-tests to recognize variances in the nurses’ COVID-19-associated stigma score. In addition, we examine the associations among age, years of working as a nurse, self-reported physical health, psychological health, social support levels, and the score of the COVID-19-associated stigma by Pearson’s correlation analyses. We also explored the factors influencing nurses’ perceived COVID-19-associated stigma by multiple linear stepwise regressions. Multicollinearity was assessed with the variance inflation factor.

## Results

### Sample characteristics

A total of 249 nurses participated in the survey, 96% (239/249) of whom were female. The mean age of participants was 31 years (SD = 5.52), and 64% (164/259) of them were married. More than half of the participants (53%) had 5–10 years of work experience as a nurse. Other socio-demographic characteristics of study participants are presented in Table 1.

**Table 1:** Sociodemographic characteristics of participants (*N* = 249).

Variables	*N* (%)	Total scores	*t/F* value	*p* value
Age (mean ± SD)	30.79 ± 5.52			
Time spent nursing (years)	9.67 ± 6.13			
Gender				
Male	10 (4.00%)	3.30 ± 2.71	0.43	0.69
Female	239 (96.00%)	2.78 ± 3.77		
Marital status				
Single	80 (32.10%)	2.71 ± 3.53	0.16	0.93
Married	164 (65.90%)	2.85 ± 3.87		
Divorced	2 (0.80%)	1.50 ± 2.12		
Domestic partnership	3 (1.20%)	3.67 ± 2.31		
Educational level (type of nursing degree)				
Certificate or associate degree	142 (57.00%)	2.54 ± 3.71	1.70	0.19
Bachelor’s degree	104 (41.80%)	3.23 ± 3.76		
Master degree	3 (1.20%)	0.33 ± 0.58		
Professional title				
Newly credentialed nurses (experience <5 years)	108 (43.40%)	2.28 ± 2.84	1.99	0.14
Experienced nurse (experience 5–10 years)	132 (53.00%)	3.17 ± 4.33		
Charge nurse (experience >10 years)	9 (3.60%)	3.67 ± 3.04		
Workplace severity of COVID-19 cases				
Severe	70 (28.10%)	2.63 ± 3.27	0.20	0.82
Mild/moderate	66 (26.50%)	3.03 ± 3.67		
Both	113 (45.4%)	2.78 ± 4.03		
Self-reported physical health (mean ± SD)	8.59 ± 1.85	/	/	/
Self-reported psychological health (mean ± SD)	8.80 ± 1.73	/	/	/
Self-reported social support (mean ± SD)	9.00 ± 1.70	/	/	/

SD, standard deviation.

### Psychometric properties of the CSI-N-3

#### Item retention

As shown in Table 2, according to the criteria of item retention, three items (I5, I12, and I14) were removed due to cross-loading (I12, I14), factor loading <0.4 (I5), and infit and outfit mean squares outside the range of 0.6–1.4 (I5, I12, and I14). After deleting I5, Cronbach’s alpha coefficient for the overall scale was increased from 0.81 to 0.84.

**Table 2:** Item analysis of the scale.

Item	Factor loading		Infit MNSQ	Outfit MNSQ	Cronbach’s *α* before removing the item^a^	Cronbach’s *α* after removing the item^b^	Item retention
	Factor I	Factor II					
I-16	0.78		0.75	0.67	0.79	0.78	Yes
I-19	0.74		0.74	0.78	0.79	0.78	Yes
I-15	0.73		0.95	0.52	0.79	0.78	Yes
I-11	0.72		0.76	0.45	0.79	0.77	Yes
I-18	0.72		0.87	1.01	0.80	0.78	Yes
I-13	0.66		0.72	0.59	0.79	0.78	Yes
I-14	0.64	0.41	1.34	0.80	0.80	0.78	No
I-17	0.61		0.96	0.83	0.79	0.78	Yes
I-9		0.76	0.80	0.51	0.80	0.79	Yes
I-6		0.74	0.73	0.39	0.80	0.79	Yes
I-4		0.69	1.54	1.00	0.80	0.79	Yes
I-2		0.69	1.04	1.44	0.80	0.79	Yes
I-1		0.54	1.57	1.33	0.80	0.79	Yes
I-8		0.53	0.97	1.06	0.80	0.79	Yes
I-12	0.48	0.51	1.55	1.72	0.80	0.79	No
I-10		0.49	0.94	0.81	0.80	0.78	Yes
I-3		0.47	1.00	0.48	0.81	0.79	Yes
I-5		0.31	2.05	2.46	0.85	0.84	No

^**^p < 0.01; MNSQ: mean squares. Factor I: nurses stigmatizing patients; Factor II: nurses being stigmatized. ^a^Before item reduction, the overall Cronbach’s α = 0.81. ^b^After item reduction, the overall Cronbach’s α = 0.84.

#### Structural validity

**Figure 2:**
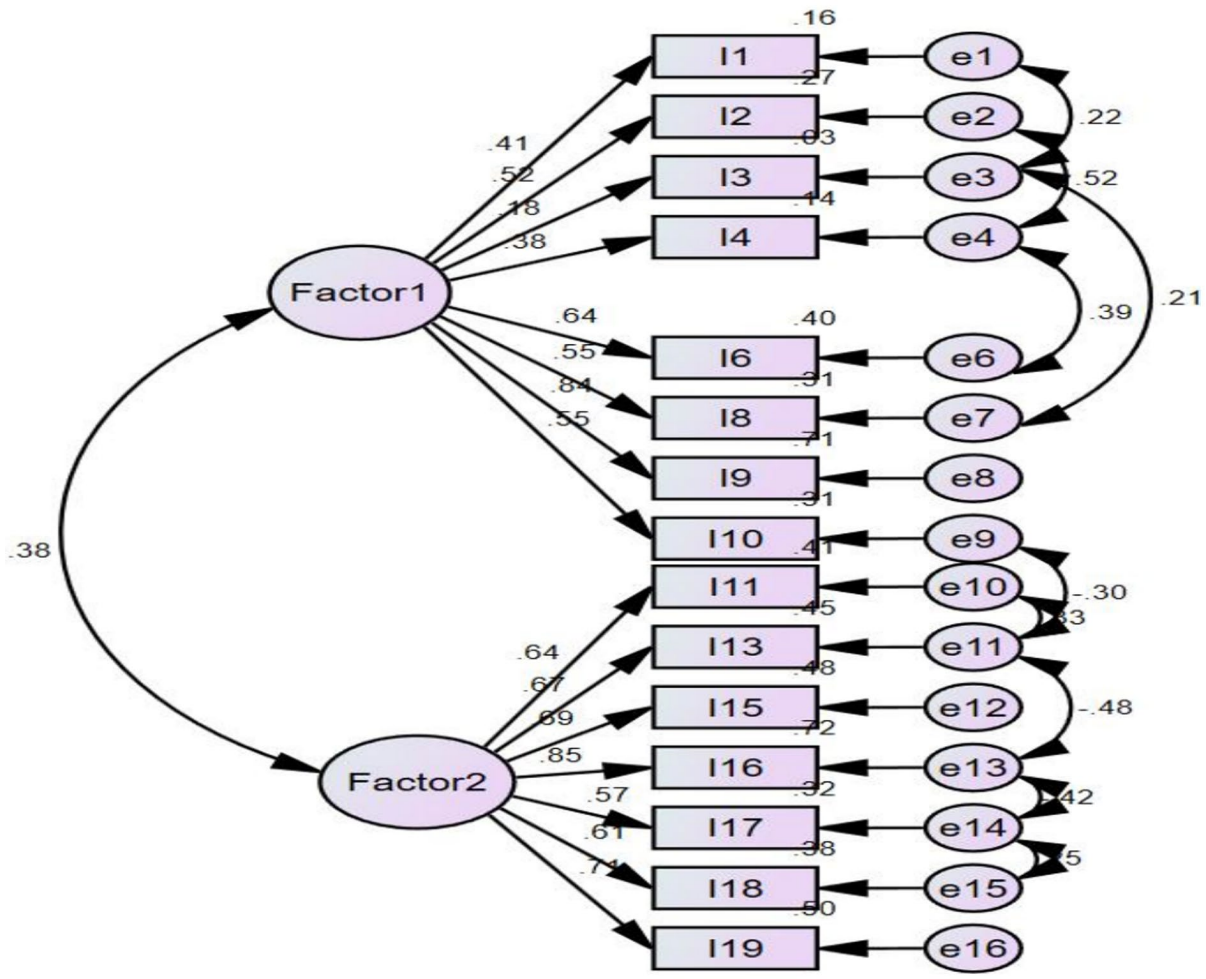
The factor structure of COVID-19 stigma instrument-nurse (CSI-N-3). Factor 1: nurses stigmatizing patients, Factor 2: nurses being stigmatized. Model fit: *χ*^2^/df (137.298) = 1.716, *p* = 0.00, RMSEA = 0.054 (95% confidence interval: 0.038–0.069), CFI = 0.955, NFI = 0.90, and TLI = 0.94.

As shown in Figure 2, the CFA showed and confirmed that the two-factor structure model demonstrated a satisfactory fit to our data [*χ*^2^/df (137.298) = 1.716, *p* = 0.00, RMSEA = 0.054 (95% confidence interval: 0.038–0.069), CFI = 0.955, NFI = 0.90, and TLI = 0.94]. Given the original structure of HASI-N, the two elements were labeled (a) nurses stigmatizing patients, and (b) nurses being stigmatized.

In the IRT analysis, the two subscale’s unidimensionality assumptions were supported by PCA; that is, the residuals explained 58.9 and 55.1% (>50%) of the raw variance, whereas the unexplained variance in the first contrast was 1.7 and 2 (<3.0) eigenvalue units.

**Figure 3:**
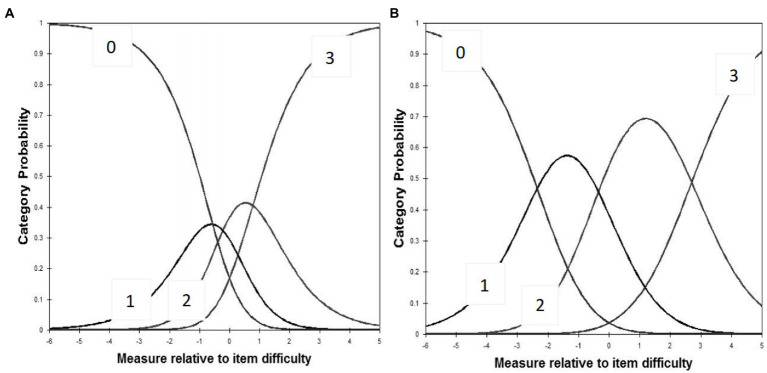
**(A)** Category probability curves for the subscale of nurses stigmatizing patients. **(B)** Category probability curves for the subscale of nurses being stigmatized. The four curves from left to right represent four response categories (0 = never; 1 = once or twice; 2 = several times; 3 = most of the time).

As shown in Table 3, the item difficulty for each item ranged from −1.77 to 1.33, and infit and outfit mean squares for each item ranged from 0.32 to 1.40. No evidence of disordered thresholds was found in the category probability curves, as the category calibration increased in an orderly way (see Figures 3A,B). During the analysis, we found the item reliability to be (0.95 and 0.96), item separation index of (4.15 and 5.12), person reliability of (0.94 and 0.94), and person separation index of (3.92 and 3.94). DIF was not found when evaluated by professional title and working place (Wolfe and Smith, 2007). Regarding the TIFs, the subscales of nurses stigmatizing patients and nurses being stigmatized gathered information most precisely when *Ө* ranged from −1.0 to 1.0 and − 2.0 to 2.0, respectively, (see Supplementary material B).

**Table 3:** The difficult, infit, outfit MNSQ, and DIF of 15 items.

Sub-scales	Item	Item difficult^a^	Infit MNSQ	Outfit MNSQ	DIF contrast by professional title^b,c^		DIF contrast by working place^d,e^	
Nurses stigmatizing patients	I-1	−0.72	1.31	1.09	0.73	0.84	−0.54	−0.45
	I-2	1.07	0.93	1.40	−0.57	−0.52	−1.05	−0.28
	I-3	−1.35	0.83	1.40	−0.20	−0.11	−0.46	−0.27
	I-4	0.68	1.25	0.67	0.48	−0.11	0.27	0.96
	I-6	0.96	0.97	0.53	1.98	2.44	1.44	1.33
	I-8	−0.20	1.01	1.37	−0.76	0.02	1.78	1.36
	I-9	1.33	0.65	0.32	0.97	0.69	1.58	0.83
	I-10	0.33	1.07	1.05	−0.49	−1.02	0.86	0.17
Nurses being stigmatized	I-11	0.53	1.04	0.87	0.15	−0.30	0.19	0.00
	I-13	0.74	0.94	0.73	−0.75	0.37	−0.69	−0.29
	I-15	1.09	1.04	0.59	1.16	−0.03	−0.38	−0.64
	I-16	−0.51	1.00	0.94	0.67	1.24	0.17	0.06
	I-17	0.08	1.40	1.22	0.24	−0.69	0.31	−0.06
	I-18	−1.77	0.96	1.00	−0.33	−0.19	0.48	0.72
	I-19	−0.15	0.90	0.88	−0.64	−0.50	−0.55	−0.33

^a^Measured in logit; positive item logit indicates that items with positive difficulties are considered to be relatively hard; while a negative item logit indicates that items with negative difficulties are considered to be relatively easy. MNSQ mean square. The DIF contrast by professional title in the following order: ^b^Naïve nurses compared with experienced nurses. ^c^Naïve nurses compared with charge nurses. The DIF contrast by working place in the following order: ^d^Severe COVID cases compared with mild/moderate COVID cases. ^e^Severe COVID cases compared with all case severities.

#### Convergent validity

The total CCSI-N-3 score was negatively correlated with self-reported physical health, psychological health, and social support levels significantly (*r* = −0.18, −0.20, and −0.21, *p* < 0.01) which was confirmed by Pearson’s correlation analysis.

#### Internal consistency reliability

The CCSI-N-3 reached Cronbach’s *α* = 0.79 (each subscale: 0.64–0.84).

### COVID-19 stigma scores of the participants

The total mean score for the CSI-N-3 in Chinese nurses was 2.80 ± 3.73 (range 0–45) overall, with a mean score of 1.42 ± 2.13 (range 0–24) for the nurses stigmatizing patients factor and a mean score of 1.38 ± 2.46 (range 0–21) for the nurses being stigmatized factor. Supplementary material C has presented the mean score for each item.

### Factors correlated with COVID-19 stigma of nurses

Self-reported physical health, psychological health, and social support levels were correlated with the score of COVID-19-associated stigma significantly (*r* = −0.18, −0.20, and −0.21, *p* < 0.05) confirmed by Pearson’s analysis. This implied that ages and years of working as nurses were not correlated with the COVID-19-associated stigma score (*r* = −0.23 and 0.01, *p* < 0.05). As shown in Table 1, other sociodemographic variables were not statistically significant (*p* < 0.05).

The total score COVID-19-associated stigma-nurse was taken as the dependent variable, and the statistically significant self-reported physical health, psychological health, and social support levels were picked as independent variables (*p* < 0.05) in the regression analysis. Regression analysis showed self-reported social support (standardized coefficients *β* = −0.206, *t* = −3.32, 95% confidence interval: −0.72 ~ −0.18) was the only factor influencing nurses’ stigma related to COVID-19, explaining 4.70% of the total variance (*F* = 5.05, *p* < 0.001). The variance inflation factor for self-reported social support was one, which is below the criteria value of 2.1.

## Discussion

### The modification and validation of the CSI-N with both CTT and IRT

This is a pioneer study to modify and validate the CCSI-N-3 *via* a thorough, multiple-The phase process. Psychometric evaluation based on the CTT and IRT demonstrated the 15-item CSI-N-3 with a two-factor solution is a trustworthy and effective self-report instrument for evaluating nurses’ COVID-19-associated stigma. The factor analytic methods used in CTT reported the equivalent factor structure model with the HSI-N (the original scale; Uys et al., 2009), including subscales of nurses being stigmatized and nurses stigmatizing patients.

In addition to the construct validity of the CSI-N, as supported by CFA, the convergent validity of the scale was also supported as there were significant negative correlations with self-reported physical health, psychological health, and social support levels. Similar to other communicable diseases such as HIV, SARS, and MERS (Park et al., 2018; Logie and Turan, 2020), our study showed that COVID-19-associated stigma adversely affects the physical and mental health of frontline nurses, although the *r* value was low. The low *r* value simply shows that these constructs were significantly correlated but different from the individual constructs (Campbell and Fiske, 1959). Importantly, Cronbach’s *α* reliability was more than 0.6, indicating that the CCSI-N-3 presented with acceptable internal consistency and reliability (Johnson et al., 2011).

Using IRT analysis, we have provided information about items in the CSI-N-3 that expand on traditional CTT methods (Adnan et al., 2018; Ahorsu et al., 2020). Our data support an ordered threshold in the category probability curves, which means that the category rating scale of the CSI-N-3 worked well and that nurses could use the scale to differentiate the four levels of item difficulty (Johnson et al., 2011; Adnan et al., 2018). The combination of a good person-separation index (>2) and person reliability (>0.8) suggests that the CSI-N-3 has acceptable measurement precision and is sensitive to distinguishing both high and low levels of COVID-19-associated stigma among frontline nurses (Johnson et al., 2011).

When represented graphically, high TIF values correlated with low standard measurement errors and therefore assure its accuracy (Hambleton et al., 1991). The most precise information provided by the TIF on the CSI-N-3 supports precise and reliable measures in the low to middle levels of the CSI-N-3. Furthermore, IRT measures also allow for the estimation of the equivalence of item calibrations across different samples and contexts (Johnson et al., 2011). In our study, we examined how 15 items may have been used differently, based on the nurses’ professional titles and the severity of cases at the workplace (mild, moderate, and/or severe COVID cases). The DIF findings showed there were no professional titles and workplace differences in the item difficulty, which further supports the stability and validity of the CSI-N-3 (Johnson et al., 2011).

### The COVID-19-associated stigma experienced by frontline nurses and its influencing factors

The score of CSI-N-3 reflects the level of COVID-19-associated stigma perpetrated or experienced by nurses; however, we found that the mean score of CSI-N-3 (2.80 ± 3.73) appears to suggest a major floor effect; that is, the level of nurses stigmatizing patients or being stigmatized was not as high as the stigma level of nurses who worked with people living with HIV [8.74 ± 9.31; (2318)] and MERS-CoV (Park et al., 2018). This finding might be explained by the cultural differences between China, where the CSI-N-3 was developed and tested, versus South Africa, where the HASI-N was developed and tested. In addition, since the original HASI-N study was conducted in 2008 before the implementation of effective interventions to decrease stigma in healthcare institutions and nursing education, the external stigma may have since decreased surrounding infectious diseases. Under the influence of Confucian culture, most Chinese nurses have manifested a greater sense of work responsibility, dedication to patient care, personal sacrifice, and professional collegiality during the pandemic (Fernandez et al., 2020; Liu et al., 2020).

During the study, milder forms of COVID-19-associated stigma were mainly noted in terms of nurses being stigmatized and gossiped about, such as being labeled as COVID-19 positive and being contagious. A possible explanation is that the general population, especially neighbors, routinely viewed nurses as a threat to the safety of others and as “disease carriers” (Hambleton et al., 1991), and thus nurses faced avoidance by community members due to this fear (Maben and Bridges, 2020). Furthermore, item 3 (*A nurse who kept her distance when talking to a COVID-19 patient*) got the highest score on the instrument, i.e., was most often endorsed. During the early months of the COVID-19 pandemic, personal protective equipment (PPEs) for nurses was in short supply and nurses knew the main perceived infection routes of COVID-19 to be by droplet, contact, and aerosols therefore they avoided close contact with patients as much as possible to protect themselves. On the other hand, even with sufficient PPE, nurses showed a certain degree of fear and stigma toward COVID-19 patients. Nevertheless, the total level of COVID-19-associated stigma was low among surveyed nurses, and nurses were unaware that their physical distancing behaviors may have biased their provision of care (Nyblade et al., 2019) and exacerbated avoidance, mistreatment, and stigma toward COVID-19 patients (Logie and Turan, 2020).

Coinciding with similar studies (Mao et al., 2018; Arshi et al., 2020), this study found that social support was negatively associated with COVID-19-associated stigma among nurses. This result suggests that social support is an effective coping strategy that can alleviate stigma. As Gardner and Moallef (2015) suggest support for nurses from the media and community as “stalwart heroism and sacrifice” contributed to their positive experiences and less perceived stigma (Gardner and Moallef, 2015). As Liu et al. (2020) indicated, multiple support systems including hospitals, colleagues, families, friends, and society can help frontline nurses minimize the stigma associated with caring for COVID-19 patients. With logistical support from their hospital, peer support, and encouragement among colleagues (e.g., the sharing of workplace experiences), frontline nurses had a sense of safety and felt less stigma (Liu et al., 2020). However, in light of the relatively small explained variance in the regression model, further exploration of other factors is encouraged, and the complexity of factors that affect COVID-19 stigma for nurses is suggested.

There are several limitations to this study. Firstly, since this study was conducted at one of the major infectious disease hospitals in Shanghai, China, it may not be representative of other Chinese-speaking areas. Secondly, the low magnitude correlations between stigma and physical health, psychological health, and social support might be due to the three single-item physical health, psychological health, and social support measures used in this study not adequately assessing these constructs. Thus, valid and reliable scales that are available in Chinese to assess nurses’ physical health, psychological health, and social support are needed to further assess the construct validity of the scale. Thirdly, using all types of social and mass media, the Chinese government is publicly encouraging all healthcare providers actively engaged in COVID-19 care. Since we recruited within an infectious disease institution in Shanghai, nurses may not have been willing to share their “true” feelings as the survey link came from their workplace. A longitudinal study is recommended to see if nurses will be more forthcoming in their answers and to compare their current and future answers to see if the passage of time and the fading of the national attention on COVID-19 will affect their responses. Furthermore, the non-significant relationship between physical and psychological health and nurses’ reported stigma may be related to measurement issues. Some CCSI-N-3 psychometric characteristics should be evaluated further, such as test–retest reliability and the responsivity or sensitivity of the CCSI-N-3, and thus, would benefit from experimental or longitudinal studies in the future. Lastly, the sample size for IRT analysis was relatively small, despite the lack of consensus on the optimal sample size. A further refinement of the scale based on testing a larger representative sample may produce more stable parameter estimates and robust results.

## Conclusion

The preliminary psychometric properties presented in this paper support the use of the 15-item CSI-N-3, which is used to measure the internal and external COVID-19-associated stigma experienced by nurses who care for COVID-19 patients during and after the COVID-19 pandemic. Although low levels of stigma in nurses were found in this study’s sample, the adverse effects of stigma during a pandemic should not be neglected. This instrument may facilitate the cross-cultural comparison of COVID-19-associated stigma experienced by nurses among different countries and expedite the improvement of additional tailored interventions for stigma reduction. Future studies should explore how to actively mobilize nurses’ social support resources to decrease the stigma associated with COVID-19 and to improve nurses’ quality of patient care and overall job satisfaction.

## Data availability statement

The original contributions presented in the study are included in the article/Supplementary material, further inquiries can be directed to the corresponding authors.

## Ethics statement

This research was approved by the relevant institutional review boards of UCLA (IRB#20–000832) and Shanghai public health clinical center (YZ-2020-S037-01). The patients/participants provided their written informed consent to participate in this study.

## Author contributions

FH and W-TC wrote the paper. FH analyzed the data. W-TC and WS designed the research study. YL and LZ performed the data collection. All authors contributed to the article and approved the final submitted version.

## Funding

This publication is a result of research supported by Fudan University Science Establishment (IDF162005), Novel coronavirus “2019-NCOV” research project of Shanghai Public Health Clinical Center (No. 2020YJKY01), Scientific research project of Shanghai Municipal Health Commission (No. 20214Y0090; PI: Sun, Wenxiu), Scientific research project of Shanghai Nursing Association (No. 2021QN-B01; PI: Sun, Wenxiu), UCLA CTSI/SON Intramural fund March 2020, and NIMH (P30MH058107; R25MH087217).

## Conflict of interest

The authors declare that the research was conducted in the absence of any commercial or financial relationships that could be construed as a potential conflict of interest.

## Publisher’s note

All claims expressed in this article are solely those of the authors and do not necessarily represent those of their affiliated organizations, or those of the publisher, the editors and the reviewers. Any product that may be evaluated in this article, or claim that may be made by its manufacturer, is not guaranteed or endorsed by the publisher.

## Footnotes

^1^ https://www.wjx.cn/jq/72175749.aspx
